# Examining Emotional Literacy Development Using a Brief On-Line Positive Psychology Intervention with Primary School Children

**DOI:** 10.3390/ijerph17207612

**Published:** 2020-10-19

**Authors:** Jacqueline Francis, Tan-Chyuan Chin, Dianne Vella-Brodrick

**Affiliations:** Centre for Positive Psychology, University of Melbourne, Parkville, VIC 3010, Australia; tanchyuan.chin@unimelb.edu.au (T.-C.C.); dianne.Vella-Brodrick@unimelb.edu.au (D.V.-B.)

**Keywords:** emotional-literacy, wellbeing-literacy, communication, wellbeing, effectiveness, positive education, implementation science, positive psychology, student-emotional-literacy

## Abstract

Wellbeing literacy (WL) may be the missing ingredient required to optimally enhance or enable positive psychology intervention (PPI) effectiveness. This study involved Victorian government funded primary schools, including two rural, two regional, and two city schools; participants included 20 classroom teachers and 131 grade five and six primary school students. A brief online PPI was implemented by teachers for 10–15 min, three times per week, for six weeks. This paper examines quantitative data collected pre and post the six week intervention, and qualitative data gathered in week one of the intervention regarding intervention effectiveness. The aim is to examine if a brief online PPI effectively builds intentional emotional vocabulary use, and to discuss how on-line PPIs can be used in public health to improve young people’s WL. Considering evaluations of process effectiveness and outcome measures related to student emotional vocabulary use, results tentatively suggest that online PPIs can positively impact emotional vocabulary capability and intentionality. Multimodal communication was exercised during the PPI, suggesting that the brief online PPI format may provide a valuable tool to promote student WL.

## 1. Introduction

An increased prevalence of mental ill-being is reported in children and adolescents [1], accounting for 16% of disease and injury in children aged 10 to 19 years [2]. This includes high rates of school-based anxiety [3] and a reduced sense of belonging [4] and significant increases in child psychiatric disorders over the last 15 years [5]. Positive psychology interventions (PPIs) are evidence-based, or evidence-informed activities, designed to protect or increase wellbeing by promoting feeling good and functioning well, and provide one pathway to enhance wellbeing [6]. Examples of PPIs within the school context include mindfulness [7], the identification and application of personal strengths [8], and gratitude practices, such as writing gratitude journals or thank you letters [9]. Access to PPIs is however variable due to barriers associated with cost (such as for training, resources, delivery, and ongoing support) as well as time [10] and social stigma [11]. Wellbeing literacy (WL) is an emerging area of research conceptualized as the mindful language use about and for wellbeing [12,13]. It is possible that WL is the missing link mediating or moderating the impact of PPIs on wellbeing outcomes [14]. In this regard, WL is an important area of study for those interested in the processes necessary to promote PPI effectiveness, with a view to the widespread dissemination of impactful wellbeing tools. PPIs themselves may also build WL. This paper examines teacher readiness, adoption, and implementation of a brief online PPI, and efficacy of a brief online tool in building WL, in the form of providing multimodal communication opportunities and in building intentional emotional vocabulary use.

WL can occur at differing degrees of sophistication and represents an intentional discourse about and for wellbeing, that involves multi-modal communication which is both receptive (can be read, heard, or viewed) and productive (can be written, spoken, or created). WL is conceptualized as having five necessary conditions [12]: vocabulary and knowledge about wellbeing, capability to comprehend multimodal wellbeing texts, capability to compose multimodal wellbeing texts, context awareness and adaptability, and intentionality for wellbeing [12]. Early measurement approaches have involved the subjective wellbeing (SWB) measure of the WellLit-6R [13] which comprises six key questions pertaining to vocabulary, knowledge, skills, comprehension, composing, and overall WL.

This paper proposes that this broad conceptualization of WL encompasses a multitude of fundamental knowledge and skills pertinent to wellbeing, including among them emotional literacy. While emotional literacy does not equate to WL, here it is considered to be a component of, or a domain of WL that is highly relevant and applicable within the school context. Emotion awareness and emotion regulation are key in social and emotional learning (SEL) curricula [15]. They are also requisite components of emotional competence (EC), emotional intelligence (EI) and emotional literacy. However, these concepts are not easily differentiated. EI stems from traditional understandings of intelligence as a fixed ability [16] and has been considered an intrapersonal intelligence [16,17]. EI can be defined as the ability to perceive, express, understand, and regulate emotions [18], with recent revisions including consideration of context, culture, and circumstance [19]. EC stems from context dependent perspectives, which are strongly tied to motivation and goal attainment [20], and developmental history [21]. EC involves having the awareness of emotions of self and others, having the capacity for adaptive coping, being context aware and responsive, and having the vocabulary to communicate emotion [20]. Emotional literacy merges and builds upon conceptualizations of EI and EC, shaped by both innate trait emotional literacy (existing ability), as well as state nurtured emotional literacy (developed skills). Emotional literacy has been defined as the ability to recognize, understand, handle, and express emotions [22] and is underpinned by conceptualizations of EI and ability [19] along with the assumption of plasticity and skill development potential [23]. Integrated with modern conceptualizations of literacy [24], an emotionally literate person may be able to perceive, understand, and express emotions, via multimodal channels, for the wellbeing of self and others, while also being responsive to context.

Previous research has shown that EC/EI are associated with positive wellbeing outcomes [25,26], including mediating wellbeing outcomes [27]. This study adds to the literature by further exploring the development of emotional literacy (as a component of WL) via a brief online PPI. These insights provide a starting place to also consider whether these literacies provide potential avenues to sustain or promote wellbeing. Do PPIs designed to promote wellbeing also build WL? If so, does WL occur before wellbeing, as a potential mediator or moderator, or simultaneously with wellbeing?

Effective PPIs have the potential to bring both habit and intentionality [28] to the learning of wellbeing vocabulary, knowledge and skills. Universal PPIs may avoid the stigma associated with targeted interventions [29], and brief online PPIs potentially overcome implementation barriers associated with cost, time, and capacity [30]. Quality implementation [31,32] may also be promoted using an online approach. Effective online PPIs hold the potential for extensive reach, positively impacting public health [33]. PPI effectiveness can be evaluated based on reach, efficacy, adoption, implementation, and maintenance considerations (RE-AIM framework: [34]). Effective PPIs are considered valuable and practical by stakeholders, demonstrate outcome success, are put into practice, adhere to key delivery requirements, and continue to be supported and used over time.

This study explores the capacity of a multi-dimensional, online, universally delivered PPI, HQthrive (hqthrive.com), to build WL in grade five and six primary school students. Specifically, this paper examines the element of emotional literacy, underpinned by EI and EC, and the specific aspect of emotional vocabulary, taught in the first week of the PPI. The four questions considered included:

1. Are there indictors of effectiveness in using a brief online PPI to build emotional vocabulary?
2. Does a brief online PPI provide students with an opportunity for multimodal communication of emotional vocabulary?
3. Are there indicators that a brief PPI can shape language use, and in particular, intentional emotional language use?
4. How might this underutilized online PPI format provide an integrated public health approach that is sensitive to context?

## 2. Materials and Methods

### 2.1. Study Design

This mixed methods study adopted an exploratory, sequential quantitative-qualitative design [35], in which qualitative data collection and analysis supplemented preliminary quantitative analysis of results. This pilot study involved the delivery of a six-week wellbeing program. The first week of the program focused on building emotional vocabulary, which is a component of emotional literacy—emotional literacy is a domain of WL. This article examines WL learning and teaching in schools using PPIs, with particular focus on the WL domain of emotional literacy learning and the sub-domain of emotional vocabulary development, informing a possible direction for future empirical studies.

### 2.2. Participants

This pilot study involved six Victorian primary schools including two rural schools, two regional schools and two city schools, drawn from a purposive sample. Twenty classes and 20 classroom teachers participated in the PPI. Data examined in this paper were collected from teachers and students who provided signed consent. This included grade five and six primary school students and their classroom teachers who completed an emotional literacy survey pre and post intervention. See Table 1 for participants providing paired data.

### 2.3. Measures

#### 2.3.1. Effectiveness—Teacher Readiness, Adoption, and Implementation

Data were gathered to examine aspects of online PPI effectiveness via Qualtrics surveys and researcher observations. Online self-report Qualtrics survey data captured teacher readiness, adoption, and implementation. Qualtrics survey data were gathered pre and post the six-week intervention, and in weekly teacher reflections. Researcher observations were recorded in field observation notes and recorded immediately following field visits during the first week of the intervention.

Questions pertaining to teacher knowledge and capacity (readiness) included: Before beginning this pilot program, how would you rate your knowledge of positive psychology, where 0 = none at all, and 10 = fully informed? Before beginning this pilot program, how would you rate your practical skills/strategies for practical application of positive psychology activities in the classroom, where 0 = none at all, and 10 = 100% comprehensive? Teachers were asked if they used the training component of the website and if so, for how long. Teachers reported how many lessons they delivered in one week, and how long they spent on the lessons. Teachers rated their overall experience with the PPI and made recommendations for changes or improvements.

Researcher observations further informed PPI effectiveness, providing information on delivery and context. The researcher directly observed implementation of week one of the online PPI at four schools, including observations of 10 teachers delivering content to 10 class groups, each visited once. The researcher recorded the number of lessons taught, time spent on lessons and technology employed, whether key messages were explicitly taught, key knowledge was shared and the extent to which students had opportunity to engage in PPI activities. The researcher gathered examples of multimodal communication as well as context sensitive feedback from students and teachers on program fit, value, and practicality. These observations were recorded in researcher journal reflections, and via survey questions, answered throughout the course of the program by teachers, and at the conclusion of the program by students.

#### 2.3.2. Outcomes—Student Emotions Language Use and Intentionality

Data were gathered via a Qualtrics student survey, to examine emotion vocabulary use. Online self-report Qualtrics survey data captured emotion vocabulary use. Qualtrics student survey data were gathered pre and post the six-week intervention. The focus on high energy emotion words was chosen to reduce any burden and contain the survey completion duration for child participants. High energy emotions and emotion regulation are also particularly pertinent within a school context, where the regulation of high energy emotions is necessary for learning and teaching. Students were asked to: Make a list of up to 10 words you know are pleasant, which are high energy emotion words; make a list of up to 10 words you know are unpleasant, which are high energy emotion words.

Language use was examined for intentionality. This idea of intentionality in language use is newly emerging and here is considered to involve desire, belief, intention, and awareness [28]. We propose an initial conceptualization of intentional vocabulary use as being total correct words minus total incorrect words. The correct use of vocabulary was interpreted as a demonstration of the desire for clear communication, belief the word fits the intended meaning, awareness of vocabulary choices, and resultant appropriate choice. For consistency, correct answers were based on the following criteria:

- Semantically correct words or phrases,
- Repeat answers by individuals were removed.
- Multiple derivatives of the base word stem were accepted.
- If the word completed the following sentences, it was deemed correct:
  ○ When I experience unpleasant high energy emotions, I feel …
  ○ After I experience unpleasant high energy emotions, I feel …
  ○ When I experience unpleasant high energy emotions, I am doing/I am with …

Intentionality scores were quantitatively analyzed, using a t-test analysis in SPSS.

### 2.4. Procedure

Focus group data collection and analysis informed the school selection process, resulting in target schools generally possessing a below average index for community, socio-educational advantage (ICSEA) score. Schools were invited from three different regions, to provide greater generalizability of results. For logistical convenience, schools within a two hour drive circumference of Melbourne were selected. Consent was provided from school principals, teachers, parents, and students prior to the collection of data. Pre-intervention data were collected in July 2018, the intervention occurred between July and September 2018, and post intervention data were collected in September 2018. This study received ethics approval from the University of Melbourne and the Department of Education and Training—Victoria.

The brief online PPI was designed to run for 10–15 min, three times per week, for six weeks across one school term, see Appendix A for an overview of the PPI. Each week aligned with a specific wellbeing focus area, for example week one focused on emotion vocabulary. Each focal category contained student learning intentions, an optional teacher training component (background), and student activities. They were designed to be sequential in nature, increasing in complexity, and involved active student participation in multi-modal tasks, see Appendix B for an example.

The brief online PPI provided students with an opportunity for multimodal communication of emotional literacy. Students were able to demonstrate their emotional literacy development by receiving and producing messages using a range of modalities, including for instance viewing videos, creating emotional vocabulary maps, and creating images of emotions.

This paper examines qualitative and descriptive data from the first week of PPI implementation, and quantitative data collected pre and post the six-week intervention. Effectiveness data relating to intentional emotional vocabulary use was analyzed quantitatively using SPSS. Effectiveness data relating to readiness, adoption and implementation were gathered via in-classroom researcher observations and self-report teacher surveys. Survey data were analyzed descriptively. Open ended questions and written researcher observations were thematically analyzed using NVIVO 12. Word cloud illustrations of vocabulary use were created using NVIVO 12. These layers of analysis created a rich illustration of online PPI effectiveness.

## 3. Results

### 3.1. Effectiveness Data—Teacher Readiness, Adoption and Implementation

#### 3.1.1. Teacher Readiness—Existing Knowledge, Skills, and Training

Teachers (*n* = 16) self-reported a range of prior positive psychology (PP) knowledge and a range of prior skill level in being able to put their PP knowledge into practice in the classroom. On a Likert scale from 0 to 10 for existing PP knowledge, teachers reported a mean of 5.5, (*SD* = 1.73) and a range from 2 to 8. For existing skills to put knowledge into practice, teachers reported a mean of 5.25, (*SD* = 1.79), and range from 1 to 8.

A teacher training component was available as part of the online PPI, for teachers to use as needed. Training components were used by at least 65% of teachers. Of survey completers 13/16 used the training component (81%). Table 2 presents information about the amount of time spent training.

#### 3.1.2. Teacher Adoption—Use of PPI

Teachers typically used the PPI for two or three days per week, with 88% of survey responders (*n* = 16) using the PPI for two or three days. Overall, teachers rated their experience using the PPI as positive. On a Likert scale from 0 = no experience at all, to 10 = a fantastic experience, survey responders scored a mean of 6.81, (*SD* = 2.24). See Table 3 for teacher self-reported experience ratings.

#### 3.1.3. Teacher Implementation—Delivery

Most teachers delivered one lesson at a time. All teachers delivered the program utilizing interactive white board technology. In three schools, teachers combined classes to team teach the PPI. Lessons ranged in length from 10–15 min (52%) to 15+ min (48%). Lessons were delivered one at a time apart from four classes. One class teacher did not deliver the PPI because they were ill. Two teachers, team teaching at one regional school delivered three lessons merged as one. One teacher at one country school ran two lessons merged as one. Teachers were mostly observed reading key messages aloud, presenting most of the key knowledge from the lesson and providing students with the time to complete activities.

### 3.2. Identified Strengths of the PPI

Team teaching of the PPI occurred at three of the six schools. Teacher delivery of online content allowed for teachers to make explicit links to curriculum and prior learnings “The teacher mentioned that she had studied poetry last term and had a word wall with emotions on it, she felt this connected and built on past class work, she felt students were receptive and understood what to do” (Researcher observation of a city teacher). Teachers also elaborated or added explanations as needed for their students “The teacher followed the program fairly closely with limited deviation and elaborated as required” (Researcher observation of a city teacher).

### 3.3. Identified Barriers and Opportunity for Future Development

While overall there was good buy-in to the PPI, it became apparent that one school with leading teacher buy-in, did not have year level classroom teacher buy-in, which resulted in extremely limited implementation and compromised fidelity (multiple lessons taught at once, key concepts overlooked or altered). The importance of both top-down and bottom-up, system wide buy-in was highlighted in this instance. Three of four teachers at this school did not complete the teacher survey. One other teacher, at another school, did not complete the teacher survey. Of note, this teacher had high absence rates during the term and rarely engaged with the PPI.

In terms of the PPI itself, teachers provided useful input used to further develop and iterate the original PPI design. The themes of task complexity and time helped shape PPI improvements. Students’ past experience and literacy capacity at the time of the PPI determined to some degree their familiarity with similes and metaphors. For some students these were new concepts, that were either difficult to understand or required more time to understand and apply. A city teacher stated that “metaphors were a difficult concept to cover in just 10 min a session”.

### 3.4. Multimodal Communication of Emotion Literacy

**Figure 1:**
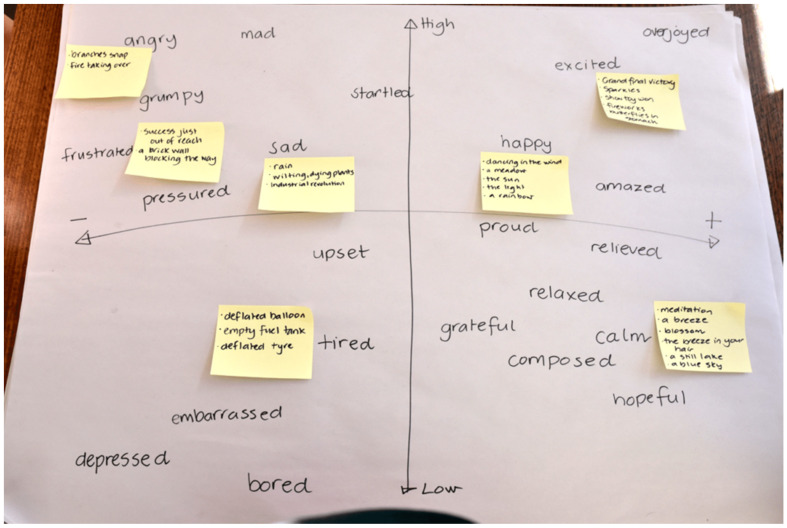
Emotions vocabulary and metaphor brainstorm.

The PPI was delivered by classroom teachers who were able to utilize the multimodal nature of the intervention for teaching and learning and for both receptive and productive communication, about and for emotional literacy. Students viewed brief information videos describing basic emotions, mapping emotions, and describing the messages in emotions. Students brainstormed, sorted, and mapped emotions, sometimes using sticky notes, sometimes writing directly onto their own emotion maps, as can be seen in Figure 1. Students viewed examples of other children’s emotional similes and metaphors, and heard audio description of these. Students listened to stories read by their teachers, sharing books and stories containing emotional metaphors in both image and text form. Students drew emotions and emotional similes and metaphors. “The visual metaphor activity was particularly engaging” (City teacher).

### 3.5. Outcomes Data—Intentional Emotional Language Use by Students

#### 3.5.1. Comparing Pleasant and Unpleasant Word Usage in Pre and Post-Measure Scores

**Figure 2:**
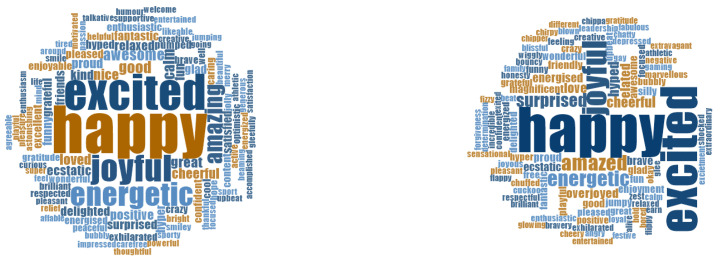
Student pleasant high energy emotion vocabulary pre- (**left**) and post- (**right**) results.

**Figure 3:**
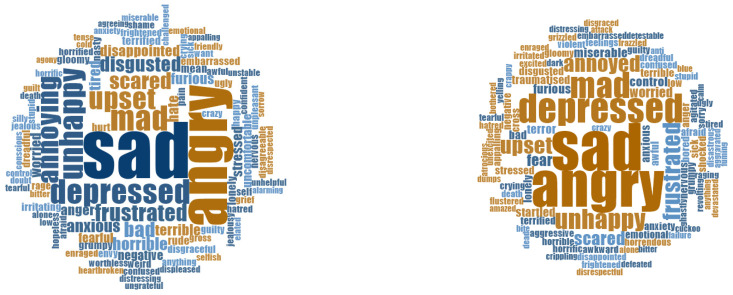
Student unpleasant high energy emotion vocabulary pre- (**left**) and post- (**right**) results.

Student vocabulary used in pre- and post-measure surveys is illustrated in Figure 2 and Figure 3. Emotional vocabulary use changed from July to September, most noticeably in the reduced error rate. While there was very little change in the percentage of students who scored 10/10, considerably fewer students scored zero at post measure scores. At pre-measurement, 15.3% of students scored zero when listing pleasant, high energy emotions, compared to 2.7% at post-measurement. At pre-measurement, 18% of students scored zero when listing unpleasant, high energy emotions compared to 7.5% at post-measurement. Fewer students made errors at post-measurement scores compared to pre-measurement scores, as can be seen in Table 4.

#### 3.5.2. Intentionality Scores

Intentionality scores were determined by subtracting the number of errors from the number of correct answers. Intentionality scores were quantitatively analyzed, using a paired t-test analysis in SPSS. These results show that overall intentionality scores were not significantly different across time, but that there was a positive trend towards increased intentionality. There was however a significant change for incorrect words used. Fewer mistakes were made across time, particularly with unpleasant high energy words. See Table 5 for more details.

## 4. Discussion

This study illustrates the effectiveness of a brief online PPI in building emotional literacy—an important domain of wellbeing literacy within educational contexts. It demonstrates the capacity of a brief online PPI in catalyzing multimodal communication and shaping language use, and in particular intentional emotional language use. Finally, it cautiously suggests how this underutilized online PPI format could provide an integrated public health approach that is sensitive to context.

This paper examines indicators of PPI effectiveness of an online multidimensional PPI, where the learning and teaching focus in week one was emotional literacy. Effectiveness data gathered during week one, as well as pre and post the six week intervention, informed understanding of readiness, adoption and implementation components of effectiveness [34]. The fit and need for context specific adaptability was highlighted in the diversity of both student and teacher capacity (teacher reported). The online PPI included teacher training components, which enabled teachers to upskill as required, promoting facilitator capacity. Online content encouraged content delivery fidelity, and teacher delivery of content allowed for flexibility for context fit. For instance, teachers were able to make links to past learning experiences such as poetry lessons. Diverse student capacity or readiness also highlighted the need to provide staged learning opportunities, meeting the learning goals of students from remembering to creating [36]. Increasing the range of learning activity complexity, in the iterative development of the PPI, provides access points for a wider cohort of students, and learning and teaching goals that more meaningfully match student readiness.

Students enthusiastically participated in the PPI and teachers mostly embraced the process, implementing the program with fidelity. PPIs deliberately designed as multimodal, hold potential to hook into existing teaching and learning capacities [24,37], building students’ understanding, knowledge, and skills within wellbeing domains, such as emotional literacy. The few exceptions to this illustrated the complex nature of field-based research. For example, classroom teachers on leave or unwell were unable to participate, and teachers at one school partially participated. This highlights the value of establishing authentic bottom up and top down buy in where possible, such that both leadership and classroom teachers consider the PPI to be acceptable.

Initial observations suggest that even a very brief PPI experience—where the PPI steps are sequenced, students are actively engaged, goals are focused and content is explicit (SAFE: [38])—has the potential to shape intentional language use, and in this instance impact emotional vocabulary used. PPIs may well promote the intentional use of language. Here a reduction in errors made over time was observed. There was also a trend toward increased intentionality. This may highlight a useful pathway to building wellbeing literacy, and potentially wellbeing itself.

EI is positively associated with skill at identifying emotional expressions among adolescents [39], possessing the vocabulary to name these expressions is necessary for the accomplishment of this skill. This study provides insight into the process of building intentional vocabulary. In previous studies, identifying and describing emotions and EI have been linked to physical wellbeing. Difficulties identifying and describing emotions have been associated with increased anxiety and decreased positive affect among university students [25], and low emotion identification skill has been associated with increased fear, decreased positive affect, and decreased social support among high school students [40]. EI has also been soundly identified as a plausible mental health, psychosomatic and physical health indicator, in two meta-analyses [41,42]. Results in this study add to existing research, demonstrating the potential of an online PPI to build emotional vocabulary in primary school aged children, a cohort that is infrequently studied. Further exploration into the development and utility of emotion vocabulary, as a component of emotional competence and emotional literacy, and as a sub domain of wellbeing literacy, including among children, is worth pursuing in future research.

The underutilized online PPI format may prove beneficial as part of an integrated public health approach. Mental health is a serious issue for young people [2]. In some cases, an inability to function effectively is not only distressing for loved ones, but drains public health resources. In Australia alone, $1.3 billion was spent on Medicare-subsidized mental health-specific benefit services, and $541 million was spent on subsidized mental health-related prescriptions during the 2018–2019 financial year [43]. Brief PPIs may be a useful piece in the puzzle. Universal, school-based interventions could help to pro-actively address some of these concerns for some students in some contexts, promoting equity such that all students can access resources irrespective of their mental health status or socio-economic-educational status. Having a brief PPI which can improve emotional literacy is a good starting point and may help erode the stigma that is often associated with mental health and wellbeing programs. Taking the ‘nudge’ approach of subtly working on WL can open the door to talking more deeply about mental health issues and may encourage young people to be more open about mental health, seeking professional help if relevant, and proactively building mental health resources.

A limit of the current study is that it did not include a control or comparison group. In addition, improvements in the number of correct emotions identified from pre- to post-program, may have occurred as a result of practice effects rather than due to the brief online PPI. Further limitations relate to the emphasis of the brief online PPI on wellbeing vocabulary literacy. Being able to identify different high energy emotions is a good start but comprehension and expression are also other important aspects of wellbeing literacy that warrant attention in future interventions and evaluations. For example, the extent to which young people can define, explain to others, and express their emotions may be a more robust determinant of wellbeing outcomes, than simply being able to identify emotions. Measuring students’ literacy for other program components such as character strengths and mindsets would also be worthwhile. Given the multidimensional nature of many PPIs and wellbeing programs, it is important to understand key areas where literacy is especially important for achieving specific wellbeing outcomes. This study is limited in that it has focused on wellbeing vocabulary for high energy emotions and does not include data to correlate this with wellbeing outcomes. It does, however, serve to prompt others to advance this area of study, particularly in the program design phase where activities could be developed with the explicit intention of building the full range of literacy across the key areas of wellbeing. Examining how wellbeing literacy influences the relationship between PPIs and wellbeing behavior using a well designed study with a control group is the ultimate goal. Other complementary studies could include qualitative methods which assess wellbeing literacy pre- and post-PPIs through focus groups, interviews, and creative assessments (digital storytelling, visual mapping, and narratives).

## 5. Conclusions

Brief online PPIs present an opportunity to build WL and wellbeing outcomes effectively and equitably. Brief online PPIs are accessible to a wide audience and they are both cost and time effective. Teacher delivery is able to uphold intervention content and delivery fidelity, while also providing flexibility for fit. Fit to context, particularly to capacity, is critical for adoption and implementation. Integrated training provides upskilling for delivery agents as required, while activities that are carefully matched to learning goals and are progressively sophisticated [36], are more likely to meet diverse learning and teaching needs.

PPIs have traditionally been designed to protect and enhance wellbeing. However, PPIs may have an additional purpose—to build WL. Measures of PPI success may therefore need to step beyond typical subjective wellbeing measures to more fully embrace evaluations of effectiveness (RE-AIM), including measures of efficacy that include WL.

This paper explored the capacity of a brief online PPI to build emotional literacy, a subset of WL. Initial school-based observations suggest that WL, and in this instance emotional literacy and language may add purpose and intent to PPI learning and teaching, providing greater clarity around the mechanisms effecting wellbeing outcomes, and insight into PPI effectiveness. Brief universal online PPIs may provide a starting equitable access point for the widespread dissemination of WL vocabulary, knowledge, and skills. These can prompt more positive attitudes towards mental health care and provide more varied, universal options that are youth friendly and feasible in schools.

## Figures and Tables

**Table 1:** Participants.

School	Number of Classes	Number of Teachers	Number of Students	Region
1	4	4	39	Country Victoria
2	3	3	18	Country Victoria
3	2	2	19	Regional
4	4	4	22	Regional
5	3	3	15	City
6	4	4	18	City

Note: Number of students listed is the number of students with signed consent who completed both pre and post emotional literacy survey data. Two additional teachers initially provided signed consent; however, these two teachers shared their classes and were not allocated to run the PPI during their teaching days. Data for these two teachers was therefore not collected.

**Table 2:** Training time of teachers self-reporting using the training component.

Time Spent Training	Number of Teachers	Percentage of Teachers
<10 min	2	15%
10–20 min	6	46%
20–30 min	4	31%
30+ min	1	8%

Note: percentages based on teachers (*n* = 13) self-reporting using the training component.

**Table 3:** Teacher rating of overall positive psychology intervention (PPI) experience.

Experience Rating	Number of Teachers	Percentage of Teachers
0	1	6.25%
1	0	0%
2	0	0%
3	0	0%
4	0	0%
5	1	6.25%
6	4	25%
7	5	31.25%
8	2	12.5%
9	1	2.25%
10	2	12.5%

Note: percentages based on teachers (*n* = 16) self-reporting. Scores range from 0 = no experience at all, to 10 = fantastic experience.

**Table 4:** Pre- and post-measurement percentages for pleasant and unpleasant high-energy vocabulary scores.

Pleasant or Unpleasant High-Energy Words	Pre- and Post-Measurement Percentages	10/10 Correct	Zero Correct	Errors Present
Pleasant high-energy words	Pre-measurement percentages	14.8%	15.3%	28%
	Post-measurement percentages	11.7%	2.7%	17.7%
Unpleasant high-energy words	Pre-measurement percentages	13.2%	18%	27%
	Post-measurement percentages	14.3%	7.5%	8.8%

Note: Post-measure *n* = 147 (78% response rate compared to pre-measurement data).

**Table 5:** T-test comparing correct and incorrect pleasant and unpleasant emotion vocabulary pre- and post-scores.

Pleasant or Unpleasant High-Energy Emotion Vocabulary	Paired Data	Mean	Standard Deviation	t	Sig. (2-Tailed)	Eta Square Effect Size (ES)
Pleasant high-energy vocabulary	Correct pleasant	0.252	3.572	0.807	0.421	0.03 small ES
	Incorrect pleasant	0.328	1.769	2.124	0.036 *	
	Total pleasant	0.580	3.863	1.719	0.088	
	Intentionality pleasant	−0.076	4.106	−0.21	832	
Unpleasant high-energy vocabulary	Correct unpleasant	0.092	3.640	0.288	0.774	0.09 moderate ES
	Incorrect unpleasant	0.443	1.382	3.667	0.000 ***	
	Total unpleasant	0.534	3.736	1.637	0.104	
	Intentionality unpleasant	−0.351	4.046	−0.993	0.322	

Note: Paired data indicated significant results for incorrect pleasant vocabulary use and incorrect unpleasant vocabulary, both reduced over time. A greater effect size was evident for change in incorrect unpleasant vocabulary use compared to incorrect pleasant vocabulary use. *** = *p* < 0.001, * = *p* < 0.05, *p* > 0.05 = non sig. *n* = 131.

## Appendix A

Overview of PPI.

Week	Focus learning and teaching area.
1	Emotions—identifying and understanding emotions e.g., building emotion vocabulary
2	Mind Body Connection—eat, sleep, move, manage stress e.g., fight, flight, freeze
3	Calm and in Control—body based strategies e.g., mindfulness
4	Perspective—mind based strategies e.g., helpful thinking
5	Character strengths—using character strengths to enhance wellbeing e.g., identifying and using personal character strengths
6	Relationships—kindness and inclusion e.g., active listening and responding in conversation

## Appendix B

Week 1 focus: Emotions.

The teacher training component included information on the Broaden and Build Theory of Positive Emotions, an article on the language of emotion, and an approach to categorizing emotions based on valence and energy.

Key teacher take away messages were:

- Emotions provide us with information about how we feel physically and emotionally, and so help with decision making.
- Emotions are tied to multi-sensory, multi-model experiences and expression.
- Pleasant emotions are beneficial to how we feel and function: positive emotions open our awareness, enhance our creativity, increase resilience and potentially improve academic performance.

Student learning objectives included building understanding that:

- Emotion words can label low and high energy feelings.
- Emotion words can label pleasant and unpleasant feelings.
- Pleasant emotions are helpful to how we feel and function.
- Our emotions can help us make wise behaviour choices.
- We understand emotions in the world around us by what we experience, for example: we watch tv, movies, dance, other people’s behaviour, we hear music, voices, sounds of nature, we touch, we smell, we feel in our hearts happiness, sadness, anger, grief, surprise and disgust.
- We communicate our own emotions by expressing how we feel in different ways: language, tone of voice, body language, dance, music, art, actions we take.

Student activities included viewing short video clips describing basic emotions, brainstorming emotional vocabulary and viewing the categorisation and mapping of emotional vocabulary. Students categorised and mapped their own emotion vocabulary as high energy or low energy and as pleasant or unpleasant. They viewed a brief video about messages in the emotions people feel and created emotions art which depicted familiar emotions.
